# Inhibition of N-glycosylation by tunicamycin attenuates cell–cell adhesion via impaired desmosome formation in normal human epidermal keratinocytes

**DOI:** 10.1042/BSR20171641

**Published:** 2018-11-28

**Authors:** Seon-Pil Jin, Jin Ho Chung

**Affiliations:** 1 Department of Dermatology, Seoul National University Hospital, Seoul 03080, Republic of Korea; 2 Institute of Human-Environmental Interface Biology, Medical Research Center, Seoul National University, Seoul 03080, Republic of Korea; 3 Deparment of Biomedical Science, Seoul National University Graduate School, Seoul 03080, Republic of Korea

**Keywords:** cell adhesion, desmosomes, keratinocytes, N-glycosylation, tunicamycin

## Abstract

N-Glycosylation affects protein functions such as location, stability, and susceptibility to proteases. Desmosomes in keratinocytes are essential to maintain epidermal tissue integrity to protect against environmental insults. However, it is not yet known whether N-glycosylation affects desmosomal functions in primary keratinocytes. Tunicamycin is an inhibitor of N-glycosylation that has been a useful tool in glycobiology. Therefore, we investigated the effect of inhibiting N-glycosylation by tunicamycin treatment on desmosomes in primary keratinocytes. In our experiments, cell–cell adhesive strength was reduced in tunicamycin-treated primary keratinocytes. TEM showed that desmosome formation was impaired by tunicamycin. Desmogleins (Dsgs) 1 and 3, which constitute the core structure of desmosomes, were well transported to the cell–cell borders, but the amount decreased and showed an aberrant distribution at the cell borders in tunicamycin-treated keratinocytes. The stability of both desmoglein proteins was also reduced, and they were degraded through both proteasomal and lysosomal pathways, although inhibiting degradation did not restore the cell–cell adhesion. Finally, tunicamycin induced desmosomal instability, enhancing their disassembly. In conclusion, these results indicate that N-glycosylation is critical to the desmosome complex to maintain cell–cell adhesive strength in primary keratinocytes.

## Introduction

In epithelial tissues, cells are anchored to one another to maintain tissue integrity to protect against the external environment [1]. There are several junctions between epithelial cells: gap junctions, tight junctions, adherens junctions, and desmosomes. Amongst these, desmosomes and adherens junctions work together to maintain epidermal cell–cell adhesion in the skin [2].

The importance of desmosomes in cell–cell adhesion is underscored in the skin by blistering disorders like pemphigus or Staphylococcal scalded skin syndrome. Hindering desmosomal function by neutralizing desmosomal cadherins (pemphigus) or by toxins (Staphylococcal scalded skin syndrome) results in significant attenuation of cell–cell adhesion [3–5], despite retaining adherens junctions. At the desmosomes, desmosomal cadherins interact with plakoglobin, plakophilin, and desmoplakin (Dsp), which are linked to the intermediate filament cytoskeleton, called keratin [6].

N-Glycosylation is an important post-translational modification of certain proteins. N-Glycosylation affects various protein functions, including structure, antigenicity, and susceptibility to proteases [7]. Tunicamycin inhibits N-glycosylation at the first step as a competitive inhibitor of N-acetylglucosamine phosphotransferase which transfers N-acetylglucosaminyl-1-phosphate to phosphorylated dolichol [8]. This property results in cytotoxicity to human cells, which precludes its application in clinical practice as an antibiotic [9]. However, tunicamycin is still vital as an N-glycosylation inhibitor in glycobiology. There have been more than a thousand publications describing the use of tunicamycin since 1973 [7].

There are a few reports describing the use of tunicamycin in dermatologic research, especially for its effects on the epidermis or primary keratinocytes [10–16]. These reports cover various effects of tunicamycin, such as inflammation, differentiation, antimicrobial peptide expression, intracellular transport, and antigenicity. However, the effect of tunicamycin treatment on desmosomes in primary keratinocytes remains to be elucidated. To answer this question, we performed a dissociation assay to measure cell–cell adhesive strength, examined the morphology of desmosome complex, and investigated the changes in desmogleins (Dsgs) resulting from tunicamycin treatment. Because desmosomes are not formed at low calcium concentrations *in vitro* [17], every assay was performed using keratinocytes incubated in a high calcium medium for 16 h.

## Materials and methods

### Cell culture

Normal human epidermal keratinocytes (NHEKs) were isolated from foreskin tissue and cultured in keratinocyte growth medium (Clonetics, Basel, Switzerland). The cells were cultured in a humidified incubator in 5% CO_2_ at 37°C. The third or fourth passage of keratinocytes was used in all experiments. Keratinocytes were serum-starved overnight in keratinocyte basal medium (Clonetics) before treatment with 100 ng/ml (120 nM) tunicamycin (Sigma–Aldrich, St. Louis, MO, U.S.A.). Human tissue for primary cultures was obtained under written informed consent of donors, in accordance with an approved protocol by the Institutional Review Board of Seoul National University Hospital.

### Dispase-based dissociation assay

Cell–cell adhesion strength of cell sheets was measured as described previously with modifications [3]. NHEKs were grown to confluence in 12-well plates. Confluent cells were incubated overnight in serum-free basal medium. Cells were pretreated with tunicamycin for 8 h and then switched to a high calcium medium (1.3 mM) with tunicamycin for additional 16 h. The cells were then incubated in 0.5 ml of PBS containing dispase II (Dispase^®^, Roche, Mannheim, Germany) for 15 min to create floating monolayer cell sheets. The floating sheets were carefully transferred to 15 ml conical tubes containing 5 ml PBS. The tubes were then subjected to mechanical stress by rotating 10–20 times in a tube rotator (Labquake™, Thermo Fisher Scientific, Waltham, MA, U.S.A.). To achieve consistent and reproducible mechanical stress, a rotator was applied instead of the manual pipetting used in another investigation [3]. PBS containing fragmented cell sheets was transferred to 35-mm culture dishes and then digitally scanned. The total number of fragments was counted by *ImageJ* software (https://imagej.nih.gov/ij/).

### TEM

Desmosome structures were analyzed using TEM images. Dispase was used to harvest the cells, while maintaining the intercellular adhesive structure. Dispase primarily disrupts cell–extracellular matrix adhesion while leaving intercellular adhesion of epithelial cells intact [18]. NHEKs were cultured until they reached confluency in 35 mm dishes. After serum starvation, tunicamycin treatment, and switching to the high calcium medium, dispase was used to release monolayer sheets. The cell sheets were transferred to 1.5 ml microcentrifuge tubes with 1 ml PBS. Harvested sheets were centrifuged at 500×***g*** for 3 min. After washing with PBS twice, the supernatant was discarded. The pellets were fixed overnight in a mixture of cold 2.5% glutaraldehyde and 2% paraformaldehyde in 0.1 M PBS (pH 7.2). The remaining procedures utilized common protocols for preparing TEM specimens.

### Western blotting

Cells were lysed in lysis buffer (1.5% SDS, 1% NP-40, 10 mM Tris, pH 8.0, and 1 mM EDTA) containing a protease inhibitor cocktail (cOmplete™, Roche). Sonication was performed and the sample was then boiled at 95°C for 6 min. Total protein concentrations were measured using BCA assay (Sigma–Aldrich). Protein samples were separated by SDS/PAGE and transferred on to PVDF membranes. After blocking with 5% skim milk in TBS containing 0.1% Tween 20, membranes were incubated with antibodies specific for desmoglein1 (Dsg1), desmoglein3 (Dsg3), tubulin, actin (Santa Cruz Biotechnology, Santa Cruz, CA), poly(ADP-ribose) polymerase (PARP) (Cell Signaling Technology, Danvers, MA, U.S.A.), ubiquitinylated conjugates (FK2 clone, Enzo Life Sciences, lnc., Farmingdale, NY, U.S.A.), GAPDH, or Dsp (Thermo Fisher Scientific). Membranes were washed using TBS with 0.1% Tween-20 and incubated with secondary antibodies. Signals were detected using an ECL solution (Supersignal West Pico, Thermo Fisher Scientific).

### Immunoprecipitation

Cells were lysed with RIPA buffer (50 mM Tris/HCl, pH 7.4, 150 mM NaCl, 0.25% deoxycholic acid, 1% NP-40, 1 mM EDTA) supplemented with protease and phosphatase inhibitors. The lysates were sonicated ten times in ice and boiled for 10 min to avoid co-immunoprecipitation with other proteins. They were precleared with 1 μg of normal IgG and protein A/G-agarose (Santa Cruz Biotechnology). Cell lysates (0.5–1 mg) were immunoprecipitated with 2 μg of Dsg1 or Dsg3 antibodies at 4°C for overnight with a tube rotator. After incubation with protein A/G agarose beads for 1 h, the immunoprecipitates were washed with RIPA buffer for five times, and eluted with 2× SDS sample buffer by boiling for 5 min.

### Immunofluorescent staining

Keratinocytes seeded on coverslips were fixed with 4% paraformaldehyde for 10 min and then permeabilized with 0.1% Triton X-100 in PBS for 8 min. The primary antibodies used were anti-Dsg1 and 3 (1:100, Santa Cruz Biotechnology) or LAMP2 (1:100, GeneTex, Hsinchu City, Taiwan). Secondary antibodies were tagged with Alexa 594® or Alexa 488® (Invitrogen).

### Statistical analysis

Statistical analyses were performed using Mann–Whitney U-test or paired *t* test. Multiple comparisons were analyzed by repeated-measure ANOVA with Tukey’s post hoc test. *P*-values of less than 0.05 were considered statistically significant. Data are expressed as mean ± S.D.

## Results

### Tunicamycin treatment decreased the adhesive strength of NHEK sheets

**Figure 1:**
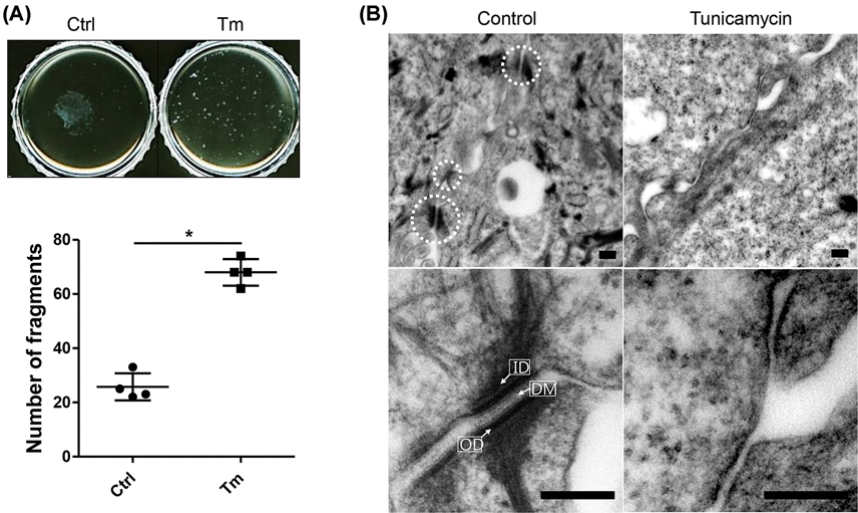
Tunicamycin treatment reduces cell–cell adhesive strength and decreases desmosome formation in differentiated NHEKs (**A**) After treatment of confluent keratinocytes with or without 100 ng/ml tunicamycin for 8 h, cells were incubated in a high calcium medium (1.3 mM) with or without tunicamycin for another 16 h. The dissociation assay was then performed. Data are depicted as means ± S.D.; *P<0.05. (**B**) Desmosomes were examined by TEM. Scale bar = 0.2 μm. Abbreviations: Ctrl, control; DM, dense midline; ID, inner dense plaque; OD, outer dense plaque; Tm, tunicamycin.

With the dispase-based dissociation assay, the total number of fragments after mechanical stress was increased in the tunicamycin-treated group compared with the control group (68 ± 4.9 compared with 25.8 ± 5.0, *P<0.05) (Figure 1A). Thus, tunicamycin decreased the cell–cell adhesive strength of NHEKs containing desmosomes. This suggests that inhibiting N-glycosylation may attenuate cell–cell adhesion.

### Tunicamycin decreased desmosome formation in NHEKs

Tunicamycin reduced cell–cell adhesive strength in differentiated keratinocytes (Figure 1A). Therefore, we examined desmosome formation under TEM to find the cause of this effect. Many well-defined standard features of desmosomes were observed at the borders of control cells: inner dense plaque, outer dense plaque, and dense midline (Figure 1B, left). However, typical features of desmosomes were rarely seen in tunicamycin-treated cells (Figure 1B, right). This implies that tunicamycin may reduce desmosome assembly or increase its disassembly, which may result in decreased cell–cell adhesive strength in NHEKs.

### Dsgs1 and 3 showed normal membrane trafficking but altered distributions at the cell borders following tunicamycin treatment

**Figure 2:**
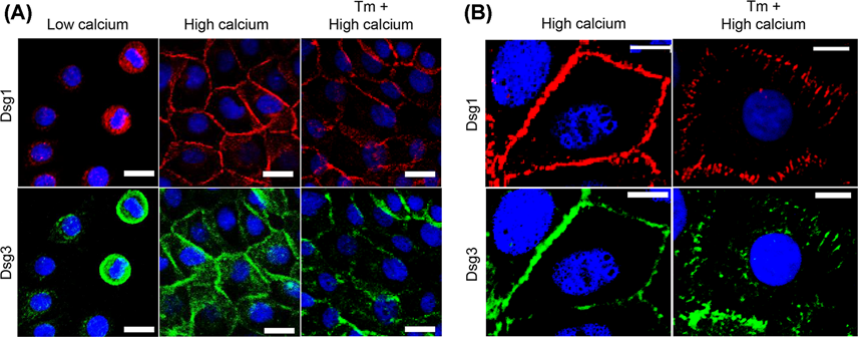
Dsgs1 and 3 show normal membrane trafficking but altered distribution at the cell borders, following treatment of NHEKs with tunicamycin (**A**) Calcium-induced membrane trafficking was examined by immunofluorescence staining. NHEKs were seeded on coverslips in a low calcium medium (0.1 mM). One day later, the culture medium was switched to the high calcium medium (1.3 mM) with or without tunicamycin pretreatment (100 ng/ml). Scale bar = 20 μm. (**B**) Distribution patterns of Dsgs were observed under high magnification. Scale bar = 10 μm. Dsg1 and 3 were labeled with rabbit polyclonal anti-desmoglein 1 (red), and mouse anti-desmogelin 3 (clone 5G11, green) antibodies, respectively. Abbreviation: Tm, tunicamycin.

Because tunicamycin led to impaired desmosome formation (Figure 1B) and Dsg1 and 3 are known to be the main adhesive proteins in primary keratinocytes [3,19], immunolocalization of Dsg1 and 3 were examined after treatment of NHEKs with tunicamycin. Dsgs are transported to the membrane through the microtubule–kinesin system for desmosome assembly [20]. Hence, membrane trafficking of Dsgs was investigated after treating NHEKs with tunicamycin. Dsg1 and 3 were localized in the perinuclear area in low calcium (0.1 mM) medium (Figure 2A). After switching to medium with 1.3 mM calcium, Dsgs were transported to the cell–cell borders, showing normal localization of Dsgs in the cells, regardless of tunicamycin treatment (Figure 2A). However, under higher magnification, an aberrant distribution of Dsgs at the cell–cell borders was observed in tunicamycin-treated cells (Figure 2B). Control cells showed a linear staining pattern of Dsgs along the cell–cell borders. In contrast, abnormal and punctate staining was frequently observed coupled with broad immunolocalization of Dsgs in tunicamycin-treated cells. Impaired desmosome assembly upon tunicamycin treatment may be attributed to this altered and abnormal distribution of Dsgs.

### The amounts and molecular weights of Dsg1 and 3 were reduced after tunicamycin treatment

**Figure 3:**
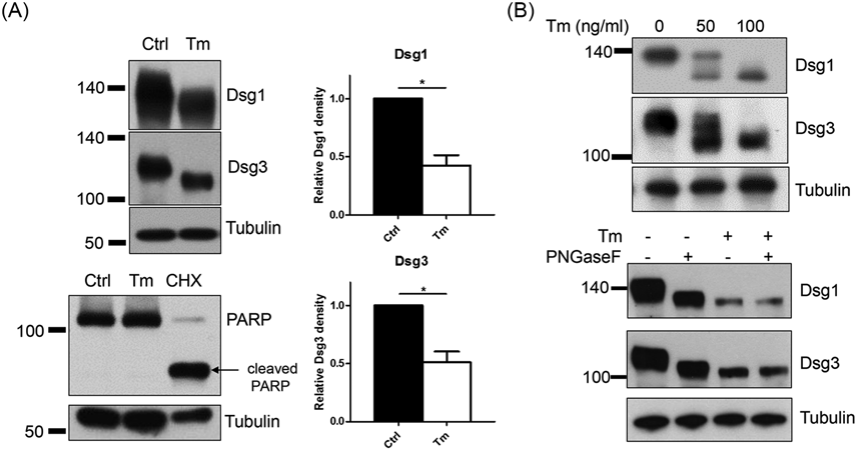
Dsg1 and 3 are reduced in amount and molecular weight, showing non-glycosylated proteins in NHEKs by treatment with tunicamycin (**A**) Confluent NHEKs were pretreated with 100 ng/ml tunicamycin for 8 h before incubating the cells in a high calcium medium (1.3 mM) with tunicamycin for an additional 16 h. Treatment with cycloheximide (100 μg/ml) for 24 h was used as a positive control for apoptosis. Western blot analyses were performed for Dsg1 and 3. Data were depicted as means ± S.D., *n*=4; *P<0.05. (**B**) Incomplete N-glycosylation inhibition was examined using different doses of tunicamycin (50 or 100 ng/ml). Deglycosylation was confirmed with PNGaseF. PNGase F reaction was performed through Bio-Rad kit according to the manufacturer’s instructions. Cell lysates were prepared with RIPA buffer. Abbreviations: CHX, cycloheximide; Ctrl, Control; Tm, tunicamycin.

Besides the immunolocalization of Dsg proteins, their expression was also investigated after treatment of NHEKs with tunicamycin. Dsg1 and 3 decreased in amount, which is consistent with reduction in the number of desmosomes. Moreover, immunoblotting for Dsg1 and 3 showed an increase in electrophoretic mobility, i.e. a decrease in their molecular sizes (Figure 3A). From this mobility shift, we found that normal Dsg1 and 3 are N-glycosylated also in NHEKs. N-glycosylation of Dsg1 and 3 in NHEKs was confirmed using dose-dependent mobility shift and PNGaseF that cleaves N-glycans from N-glycoproteins (Figure 3B). Reduced dose of tunicamycin (50 ng/ml) showed several bands of Dsgs, suggesting incomplete inhibition of N-glycosylation of Dsgs (Figure 3B). PNGase F treatment resulted in mobility shift in control cell lysate but not in a tunicamycin-treated cell lysate (Figure 3B). In this experimental setting (pretreatment with 100 ng/ml tunicamycin for 8 h and then switching to a high calcium medium (1.3 mM) with tunicamycin for an additional 16 h), NHEKs did not undergo apoptosis, as assessed by the absence of cleaved PARP. Cleaved PARP (arrow) was only positive in cycloheximide-treated cells (Figure 3A).

### Tunicamycin reduced the stability of Dsg1 and 3 proteins

**Figure 4:**
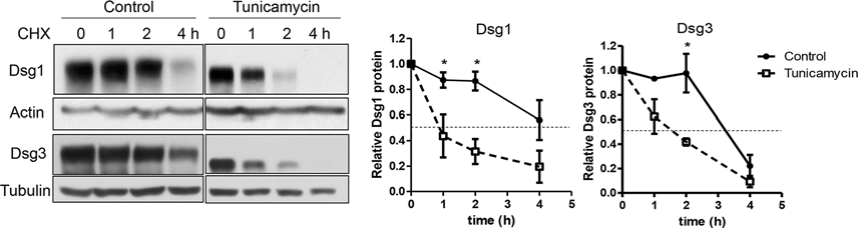
The stability of Dsg1 and 3 proteins in NHEKs is reduced by treatment with tunicamycin Confluent NHEKs were incubated in a high calcium medium (1.3 mM) for 16 h with or without tunicamycin pretreatment (100 ng/ml). Cycloheximide (30 μg/ml) was added, and the cells were harvested at 0, 1, 2, and 4 h after the cycloheximide treatment. The dashed lines indicate half the amounts of Dsg1 and 3 found at 0 h; *n*=3. Data are depicted as means ± S.E.M. Statistical analysis was performed using repeated-measure ANOVA with Tukey’s post hoc test; *P<0.05. Abbreviation: CHX, cycloheximide.

Because tunicamycin decreased the quantity of Dsgs, the degradation rate of Dsg proteins was investigated. Cycloheximide (30 μg/ml) was added for up to 4 h to inhibit protein synthesis. The reduction in protein levels was examined and the half-life of Dsg proteins was determined through protein level trend lines over time. Dsg1 in tunicamycin-treated cells exhibited a half-life of 1.7 h, while Dsg1 in control cells showed a slower degradation rate (half-life: 4.8 h). The degradation rate of Dsg3 showed similar results; the half-lives were 1.9 and 3.2 h in tunicamycin-treated and control cells, respectively. Overall, protein stability was significantly decreased for Dsg1 and 3 in tunicamycin-treated cells (Figure 4, *P<0.05)**.**

### Dsg1 and 3 proteins were degraded by both proteasomal and lysosomal pathways

**Figure 5:**
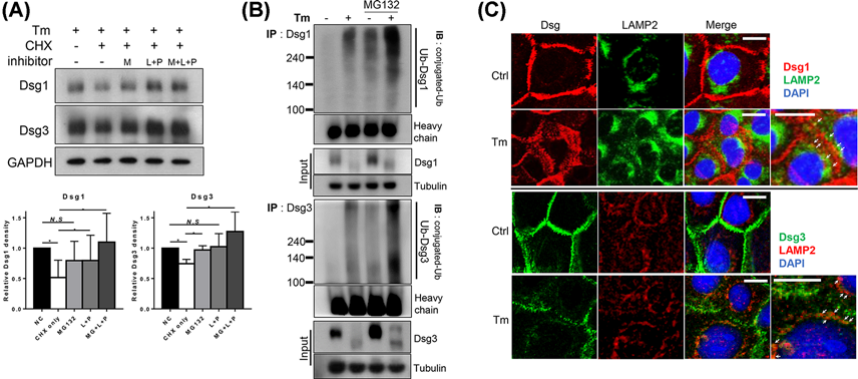
Dsg1 and 3 proteins are degraded by both proteasomal and lysosomal pathways in tunicamycin-treated NHEKs (**A**) Confluent NHEKs were pretreated with 100 ng/ml tunicamycin for 8 h then switched to a high calcium medium (1.3 mM) for an additional 16 h. MG132 (50 μM, proteasomal inhibitor), ‘pepstatin A and leupeptin (10 and 100 μM, respectively, lysosomal inhibitor cocktail)’, or both were added for 0.5 h, followed by cycloheximide (30 μg/ml). After 2 h of cycloheximide treatment, cells were harvested, *n*=4. Data are depicted as means ± S.D. Paired *t* test was used for statistical analysis. N.S, non-significant, *P<0.05. (**B**) Cells were prepared as above for tunicamycin and calcium treatment, and MG132 (50 μM) or DMSO was treated for 8 h before harvesting. Dsg1 or 3 was immunoprecipitated from cell lysates and immunoblotted for ubiquitin-conjugated proteins (poly- and mono-ubiquitination). (**C**) Cells were prepared on the coverslips. Tunicamycin and calcium were treated as above. Cycloheximide (30 μg/ml) was added at 30 min before fixation to reduce detecting newly synthesized proteins. LAMP2 was used as a lysosomal marker. Arrows indicate colocalization of desmogleins and LAMP2. Scale bar = 10 μm. Abbreviations: CHX, cycloheximide; Ctrl, control; IB, immunoblotting; IP, immunoprecipitation; M, MG132; L, leupeptin; P, pepstatin A; Tm, tucnicamycin; Ub, ubiquitin.

Dsg proteins can be degraded via various processes. Therefore, we investigated the effect of proteasomal (MG132) and lysosomal (leupeptin and pepstatin A) inhibitors on Dsg1 and 3 degradation in tunicamycin-treated cells [21]. Dsg1 and 3 levels decreased after 2 h of cycloheximide treatment. These decreases were prevented when either protease inhibitor was present (Figure 5A). To clarify the proteasomal pathway, ubiqutinated Dsgs were detected using immunoprecipitation. Ubiquitinated Dsg1 and 3 were conspicuously increased in tunicamycin-treated cells (Figure 5B). It suggests that Dsgs are ubiquitinated and degraded through proteasomal pathway in tunicamycin-treated cells. In addition to staining Dsgs in the middle of degradation pathway, cycloheximide was treated for 30 min before fixation. Punctate and broadened staining pattern of Dsgs in cell–cell borders was evident in tunicamycin-treated cells. Amongst the broadened Dsgs, those close to the cytoplasm showed colocalization with LAMP2, a marker of lysosomes (Figure 5C). Taken together, these results suggested that Dsg1 and 3 in tunicamycin-treated cells were cleared by lysosomal as well as proteasomal degradation.

### Inhibition of protein degradation did not ameliorate altered Dsg distribution or reduced cell–cell adhesive strength by tunicamycin

**Figure 6:**
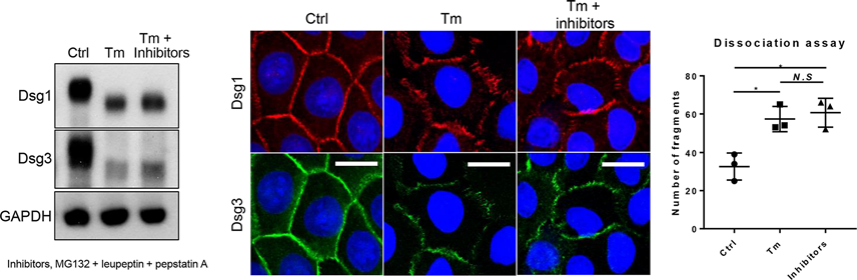
Inhibiting protein degradation does not reverse the aberrant Dsg distribution at the cell borders or reduced cell–cell adhesion by treatment with tunicamycin Confluent NHEKs were pretreated with or without 100 ng/ml tunicamycin for 8 h then switched to a high calcium medium (1.3 mM) for an additional 16 h. For protease inhibitors, MG132 (50 μM), pepstatin A 10 μM, and leupeptin 100 μM combination were treated for 4 h before harvesting or fixation. Dissociation assay was performed to measure cell–cell adhesive strength by the number of fragments. *P<0.05. Abbreviations: Ctrl, control; N.S, non-significant; Tm, tunicamycin.

Next, we wondered if tunicamycin effects were reversible by blocking protein degradation pathways. Treatment of protease inhibitors (MG132, leupeptin, and pepstatin A) increased Dsg proteins levels (Figure 6), which was also reflected in the immunostaining of Dsgs. However, inhibiting protein degradation did not alleviate the altered non-linear distribution of Dsg proteins at the cell borders. In addition, dissociation assay did not show significant changes in the number of fragments after protease inhibitor treatment (57.33 ± 6.66 compared with 60.67 ± 7.57, tunicamycin-treated only or adding protease inhibitors, respectively), either (Figure 6). These results suggest that proper distribution and forming desmosomal complexes are more important than the simple amount of protein levels.

### Tunicamycin enhanced desmosome disassembly

**Figure 7:**
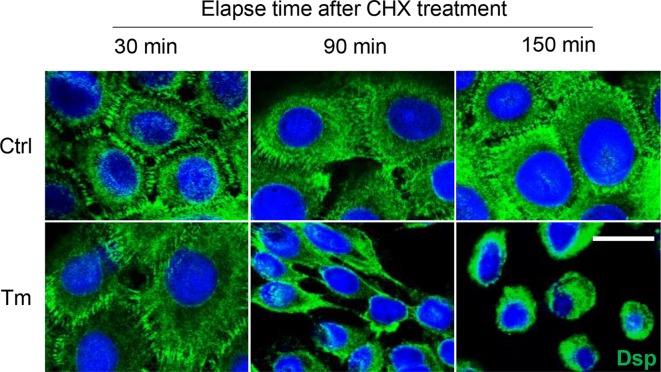
Tunicamycin destabilizes desmosomes Cells were prepared on the coverslips. Tunicamycin and calcium were treated as above experiments. Cycloheximide (30 μg/ml) was treated for indicated times (30, 90, 150 min) before fixation. Dsp, non-glycosylated desmosomal protein, was detected and visualized as green color. Scale bar = 10 μm. Abbreviations: CHX, cycloheximide; Ctrl, control; Tm, tunicamycin.

Taken together with the above results, we hypothesized that tunicamycin accelerates desmosome disassembly. After inhibiting protein synthesis using cycloheximide, immunolocalization of Dsp was examined over time (30, 90, and 150 min). Dsp is a non-glycosylated desmosomal protein, hence it would not be directly influenced by tunicamycin. As a result, Dsp was internalized to the cytoplasm after 90 min of cycloheximide treatment in tunicamycin-treated cells, whereas it still remained at cell periphery even after 150 min of cycloheximide treatment in the control cells (Figure 7). It suggests that N-glycosylation may be essential to stabilize desmosomes.

### The stability and the distribution at the cell borders of Dsp were not altered by tunicamycin

**Figure 8:**
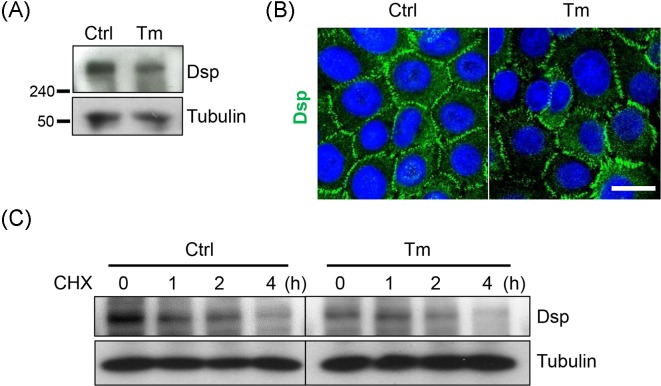
Dsp appears normal distribution at the cell borders and comparable stability despite tunicamycin treatement Confluent NHEKs were pretreated with or without 100 ng/ml tunicamycin for 8 h then switched to a high calcium medium (1.3 mM) for an additional 16 h. (**A**) Dsp was detected through Western blotting. (**B**) Cells were prepared on the coverslips. Immunostaining was performed to detect Dsp. Scale bar = 10 μm. (**C**) After treatment of tunicamycin and calcium, cycloheximide (30 μg/ml) was added, and the cells were harvested at 0, 1, 2, and 4 h after the cycloheximide treatment. Abbreviations: CHX, cycloheximide; Ctrl, control; Tm, tunicamycin.

Since Dsp showed enhanced internalization from the cell borders to the cytoplasm in tunicamycin-treated cells, we investigated the stability and distribution of Dsp protein. As expected, tunicamycin did not change the electrophoretic mobility of the protein (Figure 8A). Although the amount of Dsp protein seemed to decrease, its distribution was not altered, showing normal array pattern in tunicamycin-treated cells compared with the control cells (Figure 8A,B**)**. Finally, the stability of Dsp protein was not conspicuously compromised unlike Dsgs, either (Figure 8C). These findings imply that Dsp internalization is due to desmosome instability rather than altered characteristics of Dsp, and further that N-glycosylation is important for desmosome stabilization.

## Discussion

In the present study, we investigated the effect of inhibiting N-glycosylation with tunicamycin on desmosomes in NHEKs. Tunicamycin reduced cell–cell adhesive strength. Desmosomes, an important adhesive protein complex in differentiated keratinocytes, was not properly formed in tunicamycin-treated NHEKs. Desmosomal proteins, that comprise the core structure of a desmosome (i.e. Dsg1 and 3), were reduced in amount, and showed aberrant distribution at the cell borders, following tunicamycin treatment. The stability of Dsg1 and 3 proteins was also decreased, and Dsgs were degraded via both proteasomal and lysosomal pathways.

Desmosomes are protein complexes that must be assembled. The assembly requires sufficient protein levels of desmosomal cadherins, trafficking to the cell–cell membrane borders, and clustering to create adhesive contacts [6]. If any of these steps are abnormal, desmosome assembly becomes erroneous. Tunicamycin decreased the protein levels of Dsgs (Figure 3A). Furthermore, in the presence of tunicamycin, Dsgs were distributed aberrantly at the cell borders, although they showed normal membrane trafficking to the borders (Figure 2A,B). These aberrations (decreased protein levels and altered distribution of Dsg proteins at the cell borders) might have hampered desmosome assembly, eventually leading to reduced adhesive strength (Figure 1A,B). Another factor that might affect desmosome assembly is deglycosylated E-cadherins. E-cadherins are essential proteins that compose adherens junctions of which formation is prerequisite for the desmosome assembly [22]. In addition, N-glycans of E-cadherins are essential for the stability, folding, and trafficking of E-cadherin protein [23,24]. Therefore, deglycosylated E-cadherins produced by tunicamycin could result in reduced desmosome assembly.

Tunicamycin increased the electrophoretic mobility of Dsgs (Figure 3A). This resulted from a reduction in their molecular weight. Dsgs are considered to be glycosylated (glycoprotein) [25], and this glycosylation is now thought to involve an N-glycan [26,27]. Through reaction of PNGase F with cell lysate *in vitro*, it was confirmed that tunicamycin treatment resulted in non-N-glysocylated Dsgs (Figure 3B). These findings provide an evidence that Dsgs are N-glycosylated also in NHEKs.

N-Glycosylation regulates proper protein folding, glycoprotein quality control, endoplasmic reticulum-associated degradation, and membrane trafficking [28]. Certain proteins that are normally found as membrane-bound forms have shown intracellular retention after blocking N-glycosylation with tunicamycin [29]. However, Dsgs exhibited normal membrane trafficking in the present study, despite tunicamycin treatment (Figure 2A). These findings are consistent with the previous study shown that tunicamycin does not inhibit the transport of Dsg to the cell surface in MDCK cells [30].

Figure 3B presented that Dsgs are deglycosylated by tunicamycin treatment. Given the key role of N-glycans in proper protein folding, it seems natural that N-glycans contribute to both protein stability and structure [7,31]. Consistent with this fact, the protein stability of Dsgs was reduced by tunicamycin, as shown through changes in the protein degradation rate (Figure 4). It is plausible that deglycosylated Dsgs proteins by tunicamycin may result in misfolded proteins, decreasing protein stability. Consistent with that, Dsp, a non-glycosylated desmosomal protein, showed comparable stability in tunicamycin-treated cells with the control cells (Figure 8).

Tunicamycin increased the degradation rate of Dsgs (Figure 4). Proteins are degraded by two major pathways: the ubiquitin-proteasome pathway and the lysosomal pathway [32]. In the present study, the decreased size of Dsgs following treatment of NHEKs with tunicamycin resulted from degradation through both pathways (Figure 5). These results are consistent with the hypothesis that misfolded proteins are targetted by a combination of both proteasomal and lysosomal pathways [33].

When blocking those above protein degradation pathways, Dsgs proteins were increased (Figure 6). However, the distribution at the cell borders or ultimately the cell–cell adhesive strength was not restored by inhibiting protein degradation. These seem natural. Figure 7 showed that tunicamycin enhanced desmosome disassembly. Thus, increasing the amount of protein simply by inhibiting protein degradation in the cytosol would not increase the desmosome formation in the surface membrane of tunicamycin-treated cells. However, a previous report reads that tunicamycin treatment inhibits desmosome formation, and this was reversed by leupeptin treatment in corneal cells [34]. It should be clarified in the future studies.

At this point, unfortunately, it is not clear that N-glycosylations of which proteins are the cause of impaired desmosomal adhesion. Since there are too many N-glycosylated proteins in the cells, the results of N-glycosylation inhibition might be induced by complex processes. However, considering that the stability and the distribution of Dsp, a non-glycosylated desmosomal protein, were not altered by tunicamycin treatment (Figure 8), N-glycosylation of Dsgs proteins are likely to be important to the stability of Dsgs proteins and their normal distribution at the cell borders (Figures 2–4).

In conclusion, inhibiting N-glycosylation with tunicamycin reduces cell–cell adhesive strength via impaired desmosome formation in NHEKs. This impaired assembly might result from decreased levels of desmosomal cadherins and their aberrant distribution at the cell borders. The stability of Dsg1 and 3 proteins is also reduced, which leads to clearance of these proteins by proteasomal and lysosomal pathways. Eventually, treatment of tunicamycin enhanced desmosome disassembly. From the results of the present study, we speculate that N-glycans are important to maintain cell–cell adhesion in differentiated NHEKs by stabilizing desmosomes.

## Abbreviations

Dsg: desmoglein
Dsp: desmoplakin
NHEK: normal human epidermal keratinocyte
PARP: poly(ADP-ribose) polymerase
